# Effect of reducing the posted speed limit to 30 km per hour on pedestrian motor vehicle collisions in Toronto, Canada - a quasi experimental, pre-post study

**DOI:** 10.1186/s12889-019-8139-5

**Published:** 2020-02-10

**Authors:** Liraz Fridman, Rebecca Ling, Linda Rothman, Marie Soleil Cloutier, Colin Macarthur, Brent Hagel, Andrew Howard

**Affiliations:** 1 Departments of Paediatrics and Community Health Sciences, Cumming School of Medicine University of Calgary, Calgary, Alberta Canada; 2 Alberta Children’s Hospital Research Institute, Calgary, Alberta Canada; 3 O’Brien Institute for Public Health, Calgary, Alberta Canada; 4 Hospital for Sick Children Research Institute, Toronto, Ontario Canada; 5 Hospital for Sick Children, Peter Gilgan Centre for Research and Learning, 686 Bay St, Toronto, ON M5G0A4 Canada; 6 School of Occupational and Public Health, Ryerson University, Toronto, Ontario Canada; 7 Institut national de la recherche scientifique, Montreal, Quebec, Canada; 8 Sport Injury Prevention Research Centre, Faculty of Kinesiology, Calgary, Alberta Canada

**Keywords:** Speed limit, Pedestrian motor vehicle collision (PMVC)

## Abstract

**Background:**

Pedestrian related deaths have recently been on the rise in Canada. The effect of changing posted speeds on the frequency and severity of pedestrian motor vehicle collisions (PMVC) is not well studied using controlled quasi-experimental designs. The objective of this study was to examine the effect of lowering speed limits from 40 km/h to 30 km/h on PMVC on local roads in Toronto, Canada.

**Methods:**

A 30 km/h speed limit on local roads in Toronto was implemented between January 2015 and December 2016. Streets that remained at a 40 km/h speed limit throughout the study period were selected as comparators. A quasi-experimental, pre-post study with a comparator group was used to evaluate the effect of the intervention on PMVC rates before and after the speed limit change using repeated measures Poisson regression. PMVC data were obtained from police reports for a minimum of two years pre- and post-intervention (2013 to 2018).

**Results:**

Speed limit reductions from 40 km/h to 30 km/h were associated with a 28% decrease in the PMVC incidence rate in the City of Toronto (IRR = 0.72, 95% CI: 0.58–0.89). A non-significant 7% decrease in PMVC incidence rates were observed on comparator streets that remained at 40 km/h speed limits (IRR = 0.93, 95% CI: 0.70–1.25). Speed limit reduction also influenced injury severity, with a significant 67% decrease in major and fatal injuries in the post intervention period on streets with speed limit reductions (IRR = 0.33, 95% CI: 0.13–0.85) compared with a 31% not statistically significant decrease in major and fatal injuries on comparator streets (IRR = 0.69, 95% CI: 0.37–1.31). The interaction term for group and pre-post comparisons was not statistically significant (*p* = 0.14) indicating that there was no evidence to suggest a pre-post difference in IRRs between the intervention and comparator streets.

**Conclusions:**

Declines in the rate of PMVC were observed on roads with posted speed limit reductions from 40 km/h to 30 km/h, although this effect was not statistically greater than reductions on comparator streets.

## Background

In 2015, pedestrians accounted for 15% of all road traffic-related deaths in Canada [1]. In addition, a 2011 OECD report noted that Canada was one of only seven industrialized nations where pedestrian-related deaths had increased over time [2, 3]. In the City of Toronto, over the 12-year period 2005 to 2016, inclusive, 2172 pedestrians were killed or seriously injured after being struck by a motor vehicle [4].

Lower speed roads are associated with a reduced risk of pedestrian motor vehicle collision (PMVC) as well as less severe PMVC injuries [5]. A comprehensive literature review that examined pedestrian fatality risk as a function of car impact speed showed that for every 1.6 km/h reduction in speed, PMVC frequency was reduced by 5% [6]. The chance of surviving a collision with a motor vehicle traveling at 50 km/h is less than 20%; whereas, survival increases to 50% at 40–45 km/h and 90% at 30 km/h [1]. Globally, studies in South Africa, New Zealand, Europe, and North America have shown that average vehicle speeds are reduced by 8–40% after speed limits are lowered from 60 km/h to 50 km/h [7]. A meta-analysis of impact speed and pedestrian fatality risk supports setting speed limits of 30–40 km/h for high pedestrian activity areas as the risk of a fatality reaches 5% at an estimated impact speed of 30 km/h [8]. Although many studies report a reduction in severe PMVC injuries and crash risk after lowering speed limits, speed limit reductions have not been well studied using controlled quasi experimental designs [5, 9, 10].

Reasons for the increased likelihood of a crash at higher speeds include a reduced field of vision, shorter reaction time, and increased stopping distance once the brakes are engaged. At lower speeds, pedestrians can make more effective decisions about when to cross the road and drivers have sufficient time to stop [11]. Toronto’s Vision Zero Road Safety Plan has proposed posted speed limit reductions from 40 km/h to 30 km/h on local roadways in an effort to reduce severe and fatal PMVC injuries [4, 12]. However, there are few pre-post studies that examine the effect of such speed limit reductions on PMVC. The aim of this study was to examine the effect of reducing posted speed limits from 40 km/h to 30 km/h on incident rates of PMVC in Toronto, Canada.

## Methods

### Study design

A quasi-experimental, pre-post study design with a comparator group was used. Ethical approval of the study was obtained from the Hospital for Sick Children Research Ethics Board (REB#: 1000059622).

### Intervention

**Fig. 1:**
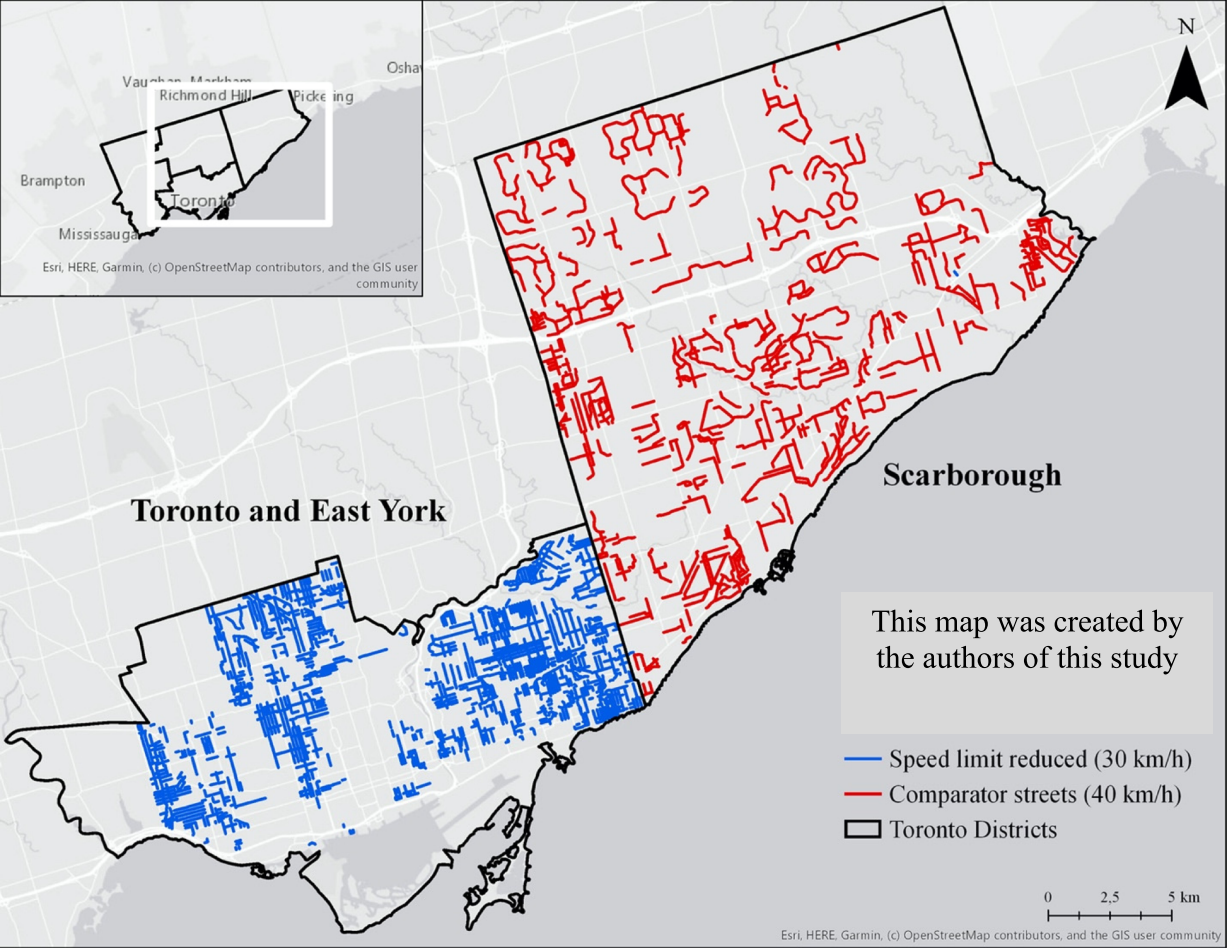
Map of 30 km/h Speed Limit Reduced Streets and 40 km/h Comparator Streets in Toronto. Intervention streets with speed limit reductions from 40 km/h to 30 km/h are shown in blue and are mainly located in the Toronto and East York District. Comparator streets that remained at a 40 km/h posted speed limit are shown in red and were selected from the Scarborough District. Speed limit reductions were applied to all neighborhood roads in Toronto and East York district, irrespective of the actual collision frequency

Between January 2015 and December 2016, [12] the City of Toronto used a blanket approach and reduced the posted speed limit from 40 km/h to 30 km/h on all local roads within 12 Municipal Wards in the Toronto and East York District (see Fig. 1) [13]. The location of streets with speed limit reductions and the dates of implementation of the intervention were obtained from the City of Toronto, Transportation Services Division. Street names were used to identify street segments within a map of the Toronto road network, (using ArcMap 10.5.1) to determine the number and length of street segments that had posted speed limit reductions.

### Comparator streets

Comparator streets were selected in the Scarborough District where local roads with posted speed limits of 40 km/h remained unchanged throughout the study period, between January 2013 and December 2018 (see Fig. 1). Data on posted speed limits in the City of Toronto were obtained from a publicly available database [14]. Comparator streets were included if the speed was marked as “confirmed” and “no change” in the dataset. Last, a data quality check was performed on 20% of the streets to confirm that streets had a 40 km/h posted speed limit throughout the entire study period. This was completed using Google Street View, which provided historical data from 2013 to 2018 to verify 40 km/h posted speed limit signs.

### Outcome data

Toronto Police Service data were used to identify all PMVC from January 2013 to December 2018. If there was insufficient information in the police report to confirm where the collision occurred, the PMVC was excluded from the analysis. All PMVC were then mapped and assigned to either a “speed limit reduced street segment” or “comparator street segment”. PMVC that occurred within an intersection (+/− a 25-m zone surrounding the intersection) were included if: [1] the direction of travel; and/or [2] the road classification on the police report indicated that the PMVC occurred on a speed limit reduced street or comparator street. Since speed limit reductions occurred in 2015 and 2016, PMVC data on speed limit reduced street segments were available for a minimum of 2-years pre-, and 2-years post-implementation of the intervention. For comparator streets, PMVC data were available for 3 years pre- and post- based on the midpoint of the study period.

For descriptive purposes and stratified analyses, additional information on the police reports that were obtained included: pedestrian age, injury severity, and collision location (intersection or midblock), as well as environmental conditions (traffic control devices, visibility, lighting, and road surface). Pedestrian age was categorized as child (0 to 15 years), adult (16 to 59 years) and older adult (60 years and over). Injury severity was classified in the police reports as: no injury, minimal injury (no medical attention); minor injury (seen in an emergency department); major injury (hospital admission); and fatal injury. For this study, minimal and minor injuries were combined into a single category as these types of injuries are commonly misclassified in police reports. Major and fatal injuries were also combined into one category because of the small numbers in each category. Environmental conditions were classified as: traffic control devices (none or any), visibility (clear or other), lighting conditions (daylight or other), and road surface conditions (dry or other).

### Co-interventions

Information on other speed-related co-interventions on local roads was also gathered to account for potential bias. This included information on senior safety zones, flex-post signs, red light cameras, watch your speed boards, and school safety zone interventions (such as pavement markings, flashing beacons, school signage, and zebra crosswalks). Spatial coordinates of the co-intervention locations were obtained from the City of Toronto Open Data Catalogue [15]. ArcGIS, was used to identify the number of speed limit reduced and comparator street segments that had senior safety zones, flex-post signs, red light cameras, and watch your speed boards, as well as those within 250-m of schools with implemented safety zone interventions.

### Statistical analyses

All statistical analyses were conducted using SAS V.9.4. To evaluate the effect of reducing posted speeds from 40 km/h to 30 km/h on PMVC rates, we compared the rate of PMVC on street segments before and after the intervention (and against comparator streets where posted speed limits remained at 40 km/h) using repeated measures Poisson regression. A similar statistical approach has previously been used to study the effects of other traffic safety interventions such as street car right of way, crossing guards, pedestrian countdown signals, and speed humps [16–19]. Generalized estimating equations with an autoregressive correlation structure were used to fit the models to account for repeated observations of the roadway segments. PMVC rates were calculated as the number of collision events per 100 km per month. A pre-post predictor variable was created to categorize collisions that occurred before the installation date (pre) and after the installation date (post). Collisions that occurred on the same day of the installation were excluded because installing new speed limit signs may have affected vehicle travel speeds and PMVC risk. For comparator streets, since there was no reduction in posted speed limits, we used the midpoint date of the study period (i.e. January 1, 2016) to define the pre and post period for the analysis. To take into account seasonal effects which have been reported in other studies [18, 19], the model included season of collision, categorized as summer (April to September) and winter (October to March). We ran stratified models for speed limit reduced streets (Model 1) and comparator streets (Model 2) to evaluate separate pre-post comparisons in each group. Additional analysis of PMVC by age, injury severity, location, traffic control devices, visibility, lighting, and road surface conditions were examined using similar stratified models for both intervention and comparator groups. A model including all intervention and comparator streets together was estimated including an interaction term for the pre vs post and intervention vs. comparator group comparisons. Lastly, sensitivity analyses which excluded streets with co-interventions were performed for both intervention and comparator groups. Incidence rate ratios (IRRs) – adjusted for season - were reported with 95% confidence intervals (CIs). Statistical significance was set a priori at 0.05.

## Results

### Intervention and comparator streets

In 2015 and 2016, posted speed limits were reduced from 40 km/h to 30 km/h on a total of 850 roadway segments comprising 303.8 km in the City of Toronto. The majority of roads received the intervention in 2016 (68%) and between September and November (72%).

A total of 390 roadway segments in the Scarborough District were included as comparator streets. These comparator streets remained at a posted speed limit of 40 km/h during the entire study period and spanned 289.5 km in the City of Toronto.

### Pedestrian-motor vehicle collisions

From January 2013 to December 2018, a total of 10,624 PMVC were reported in the City of Toronto of which, 230 were fatal. From 2013 to 2018, on average, the annual incidence of PMVC decreased by approximately 1% (1731 to 1718, respectively). During the time period of study, there were 382 PMVC that occurred on 850 speed limit reduced roadway segments and 292 PMVC that occurred on 390 comparator street segments.

Table 1 shows the frequency of PMVC by implementation of the intervention (pre, post) and by season (summer, winter) along with IRRs and 95% CIs for intervention and comparator streets. Table 2 shows PMVC frequency, by age, injury severity, location, and environmental conditions along with IRRs and 95% CIs for intervention and comparator streets.

**Table 1:** Frequency and incidence rate ratios of PMVC for intervention and comparator groups

Models	Covariate	PMVC	Total Km-Month	PMVC per 100 Km-Month	Adjusted IRR^a^ (95% CI)
Model 1 Intervention Streets	Pre-Speed Limit Reduction	245	12,286	1.99	1
	Post-Speed Limit Reduction	137	9600	1.43	0.72 (0.58–0.89)
	Summer	194	10,970	1.77	1
	Winter	188	10,915	1.72	0.98 (0.79–1.21)
Model 2 Comparator Streets	Pre-Speed Limit Reduction	151	10,428	1.45	1
	Post-Speed Limit Reduction	141	10,437	1.35	0.93 (0.70–1.25)
	Summer	111	10,456	1.06	1
	Winter	181	10,409	1.74	1.64 (1.27–2.12)

^a^based on full model, with pre-post and season covariates

**Table 2:** PMVC Incidence Rate Ratios (IRR) and 95% Confidence Intervals for Intervention and Comparator Streets, Stratified by Collision Characteristics

Characteristics	Intervention Streets (*n* = 382 PMVC)		Comparator Streets (*n* = 292 PMVC)	
	Total n (%)	IRR^a^ (95% CI)	Total n (%)	IRR^a^ (95% CI)
Ages				
Child (0 to 15)	50 (13.1%)	0.72 (0.41–1.29)	43 (14.7%)	0.79 (0.40–1.57)
Adult (16 to 59)	235 (61.5%)	**0.71 (0.55–0.92)**	187 (64.0%)	0.99 (0.71–1.38)
Older Adult (Over 60)	89 (23.3%)	0.79 (0.51–1.23)	60 (20.5%)	0.87 (0.51–1.49)
Unknown	8 (2.1%)	–	2 (0.68%)	–
Injury Severity				
None	18 (4.7%)	0.51 (0.18–1.45)	10 (3.4%)	0.25 (0.05–1.32)
Minimal and Minor	330 (86.4%)	**0.78 (0.62–0.98)**	238 (81.5%)	1.03 (0.75–1.42)
Major and Fatal	34 (8.9%)	**0.33 (0.13–0.85)**	44 (15.1%)	0.69 (0.37–1.31)
Location				
Intersection	310 (81.2%)	**0.62 (0.48–0.79)**	255 (87.3%)	0.92 (0.68–1.24)
Midblock	64 (16.7%)	1.01 (0.64–1.60)	31 (10.6%)	0.72 (0.33–1.59)
Other	8 (2.1%)	–	6 (2.1%)	–
Traffic Controls				
No Control	171 (44.8%)	**0.63 (0.45–0.87)**	73 (25.0%)	0.87 (0.49–1.54)
Any Traffic Controls	210 (55.0%)	0.78 (0.60–1.03)	219 (75.0%)	0.95 (0.67–1.33)
Unknown	1 (0.2%)	–		
Visibility				
Clear	319 (83.5%)	**0.69 (0.54–0.87)**	257 (88.0%)	0.98 (0.71–1.34)
Rain, Snow or Other	63 (16.5%)	0.88 (0.54–1.41)	35 (12.0%)	0.66 (0.33–1.36)
Lighting				
Daylight	258 (67.5%)	**0.69 (0.53–0.90)**	181 (62.0%)	0.83 (0.57–1.21)
Dawn, Dusk & Dark	124 (32.5%)	0.77 (0.53–1.12)	111 (38.0%)	1.13 (0.74–1.72)
Road Surface				
Dry	286 (74.9%)	**0.73 (0.56–0.94)**	233 (80.0%)	1.01 (0.72–1.40)
Other	96 (25.1%)	0.68 (0.46–1.00)	59 (20.0%)	0.68 (0.40–1.16)

^a^adjusted for season

IRR and 95% CI significant at 0.05 are bolded

As shown in Table 1, the PMVC per 100 km per month was 1.99, pre-intervention (i.e. at posted maximum speeds of 40 km/h) and 1.43, post intervention (i.e. at posted maximum speeds of 30 km/h). This represents a statistically significant 28% decrease in the PMVC rate following implementation of the intervention, after adjusting for season (IRR post- versus pre-: 0.72, 95% CI: 0.58–0.89). There was no material difference in PMVC rate between summer (1.77/100 km/month) and winter (1.72/100 km/month); IRR winter versus summer (0.98, 95% CI: 0.79–1.21). For comparator streets (i.e. streets that remained at 40 km/h speed limits throughout the period of study), the PMVC per 100 km per month was 1.45 in the pre-period and 1.35 in the post period. This represents a 7% decrease in PMVC rate after adjusting for season; however, this decrease was not statistically significant (IRR post- versus pre-: 0.93, 95% CI: 0.70–1.25). There was a significant difference in PMVC rate between summer (1.06/100 km/month) and winter (1.74/100 km/month). The PMVC rate significantly increased by 64% in the winter months (October to March) (IRR winter versus summer: 1.64, 95% CI: 1.27–2.12).

For both intervention and comparator streets, the majority of PMVC involved adults between the age of 16 and 59 years (61.5% vs. 64.0%) and minimal and minor injuries (86.4% vs. 81.5%). Similarly, the majority of collisions occurred at an intersection (81.2% vs. 87.3%), in areas where a traffic control device was present (55.0% vs. 75.0%), in clear conditions (83.5% vs. 88%), in daylight (67.5% vs. 62.0%) and on dry roads (74.9% vs. 80%).

As shown in Table 2, following implementation of the intervention, decreases in PMVC rates on speed limit reduced streets remained apparent in different collision sub-groups, with observed PMVC rate reductions by 29% among adults (IRR: 0.71, 95% CI: 0.55–0.92), and 38% at intersections (IRR: 0.62, 95% CI: 0.48–0.79). Likewise, PMVC rates significantly declined (post- versus pre-) in clear conditions, in daylight, and on dry roads. Most notably, there was a 67% decline in major and fatal injury frequency post- versus pre-implementation (IRR: 0.33, 95% CI: 0.13–0.85), which was statistically significant. Among comparator streets, although no significant changes were observed, the largest decreases in PMVC rates were seen in children (IRR = 0.79, 95% CI: 0.40–1.57), no injuries (IRR = 1.03, 95%CI: 0.75–1.42), in midblock locations (IRR = 0.72, 95% CI: 0.33–1.59), collisions with no traffic controls (IRR = 0.87, 95% CI:0.49–1.54), conditions with unclear visibility (i.e. rain, snow or other) (IRR = 0.66, 95% CI: 0.33–1.36), in daylight conditions (IRR = 0.83, 95% CI: 0.57–1.21) and on wet or other road surface conditions (IRR = 0.68, 95% CI: 0.40–1.16).

When estimating a model including the interaction term for group and pre-post comparisons, the interaction term was not statistically significant (*p* = 0.14) indicating that there was no evidence to suggest a pre-post difference in IRRs between the intervention and comparator streets. From that model, the IRR for the post-pre comparison for the comparator group was 0.93 (95% CI: 0.70–1.25) and for the intervention group it was 0.71 (95% CI: 0.57–0.88).

### Sensitivity analyses

To consider potential bias of other co-related speed interventions implemented in the City of Toronto on PMVC rates, sensitivity analyses were performed on roadway segments with no identified co-interventions. For speed limit reduced streets, co-interventions were found on 2.4% of segments (*n* = 21) including school safety zone interventions (*n* = 11), watch your speed boards (*n* = 9), senior safety zones (n = 2), and red-light cameras (n = 1). Two street segments were found to have more than one of the identified co-interventions; all other street segments contained only one co-intervention. After excluding the 21 street segments with co-interventions, results of the sensitivity analysis were comparable to the main analysis, with a similar IRR for PMVC following speed limit reductions (IRR post- versus pre-: 0.70, 95% CI: 0.56–0.87).

For comparator streets, co-interventions were located on 4.1% of comparator segments (*n* = 16) which consisted of school safety zone interventions (*n* = 10), red-light cameras (*n* = 3), flex-post signs (*n* = 2) and watch your speed boards (n = 1). Findings from the sensitivity analysis which excluded the 16 comparator segments with co-interventions were congruent with the main analysis (IRR post- versus pre-: 0.91, 95% CI: 0.66–1.23).

## Discussion

A reduction in the posted speed limit from 40 km/h to 30 km/h was associated with a 28% decrease in PMVC incidence rates in the City of Toronto between 2013 and 2018, while there was a non-significant 7% decrease in PMVC on streets with unchanged 40 km/h speed limits. This protective effect was significant among adults 16 to 59 years of age and at intersections on speed limit reduced streets. Speed limit reduction also had a strong impact on PMVC injury severity: the percentage of major and fatal PMVC injuries decreased significantly by 67% in the post-intervention period.

The intervention to reduce posted speed limits to 30 km/h in the City of Toronto was implemented on all 40 km/h local roads in the Toronto and East York Districts, and was not based on historical PMVC frequency [12]. Therefore, “regression-to-the-mean” is an unlikely explanation for the effect noted. The effectiveness of posted speed limit reduction on PMVC risk and PMVC injury severity found in this study is similar to previously reported studies [20–23].

The interaction term between group and pre vs post comparison was not statistically significant, indicating no statistical evidence for effect measure modification of the pre-post comparison by group. However, the magnitude of the reduction in the PMVC rate was greater in the intervention group and the confidence limits did not include the null value. Interaction tests can be underpowered and this is what may have influenced our results. In any case, the pre-post comparison for the intervention group is in line with the effect estimates from other investigations of reduced speed limits [21].

### Crash risk reduction and injury severity

Higher vehicle speeds increase the likelihood of PMVC and serious injury [24]. Pedestrians struck by a motor vehicle travelling at 50 km/h are 8 times more likely to die, compared with pedestrians struck by vehicles traveling at 30 km/h [25]. A systematic review on the relationship between impact speed and the probability of pedestrian fatality during a vehicle-pedestrian crash found that on average, when the estimated impact speed increases by 1 km/h, the odds of a pedestrian fatality increases by 11% [26]. In the UK, a 20-year time-series study showed that 20 mph (32 km/h) zones were associated with a 42% reduction in road casualties [20]. A study that examined lowering speed limits from 90 km/h to 70 km/h on highways in Belgium found a 5% decrease in crash rates and a 33% decrease in serious injuries and fatalities when speed limits were reduced [21]. A 2018 study from Edmonton, Canada found a 45% reduction in fatal and severe injury collisions when speed limits were reduced from 50 km/h to 30 km/h in school zones [27]. In addition, injuries to vulnerable road users (pedestrians and cyclists) were reduced by over half (55%) [27]. Another study from New York City reported that road casualties dropped by 8.74% after implementation of neighborhood slow zones (20 mph speed limit areas) in 2011 [9]. This study also found a cost-savings of $15 USD per resident to the healthcare system when slow zones were in place. Although all of these studies reported a decrease in PMVC following speed limit reductions, these streets were not compared to other streets where the limits remained unchanged. The findings in this study were consistent with other jurisdictions and further add to the literature by demonstrating a larger significant reduction of PMVC on speed limit reduced streets compared to a small non-significant decrease on comparator streets.

### Strengths

A major strength of this study is the controlled pre-post design, which compared PMVC rates on the same streets where speed limits were decreased from 40 km/h to 30 km/h to the PMVC rates on comparator streets. The large magnitude of the effect on treated streets (28%) compared with the small, not statistically significant decrease in pedestrian collisions on comparator streets (7%) suggests that the effect is likely due to the intervention.

Additionally, the pre-post design of this study allowed for comparison of the intervention (40 to 30 km/h speed limit reductions) while controlling for other features within the built environment. In addition, other speed reduction co-interventions such as senior safety zones, flex-post signs, red light cameras, watch your speed boards, and school safety zone interventions (pavement markings, flashing beacons, school signage, and zebra crosswalks) were considered in the analysis and were congruent with the main analysis for both intervention and comparator streets.

### Limitations

The largest limitation of this study is the lack of vehicle and pedestrian volume (exposure) data. The City of Toronto does not regularly collect pedestrian counts (exposure data) or traffic volumes on local streets. Reduced vehicle speeds may increase pedestrian volume, but would be unlikely to decrease pedestrian volume and so the local safety effect is likely conservatively estimated. If vehicles are displaced to neighboring roads due to speed reductions, then area wide effects may occur that would be inapparent in our study design.

Public health officials and other municipal stakeholders should consider collecting pedestrian and vehicle volume data in the future to strengthen our understanding of reductions in PMVC rates.

A second significant limitation is the suitability of the comparator group of streets. The intervention was not designed as an experiment, and the intervention was applied across an entire area of the city which is not identical geographically or socially to any other area. While the presence of a group for comparison is a relative strength of the study, the only way to ensure comparability of intervention and comparator streets is through prospective experimental design which is rare in traffic injury research.

For this study the post-intervention period consisted of at least 2 years of data; therefore, longer-term effects of posted speed limit reductions could not be determined. The short-term effect of a speed reduction may be enhanced due to novelty and/or enforcement. Although speed limit reduction showed an overall effect on all PMVC, the study had low statistical power to detect differences by specific collision characteristics, e.g., age, injury severity because of small sample sizes within stratified groups. Last, the frequency of PMVC differs by road type. A report published by the City of Toronto, Transportation Services found that only 8% of collisions in the Toronto and East York District occurred on local roads, compared with 63% on major arterials and 24% on minor arterials from 2009 to 2013 [13]. Therefore, the findings from this study should be generalized only to local roads with speed reductions from 40 km/h to 30 km/h.

## Conclusions

In Toronto, important declines in the rate of PMVC were observed on roads with posted speed limit reductions from 40 km/h to 30 km/h, although this effect was not statistically greater than reductions on comparator streets. Longer-term outcomes of posted speed limit reductions in Toronto need to be further studied.

## Data Availability

The data that support the findings of this study are available from the City of Toronto, Transportation Services but restrictions apply to the availability of these data, which were used under license for the current study, and so are not publicly available. Data are however available from the authors upon reasonable request and with permission of the City of Toronto.

## Abbreviations

CIs: Confidence i`ntervals
IRRs: Incidence rate ratios
PMVC: Pedestrian motor vehicle collision
